# Simultaneous Adsorption and Purification of Low-Concentration SO_2_ and H_2_S

**DOI:** 10.3390/molecules30112302

**Published:** 2025-05-24

**Authors:** Xiaoli Cao, Lin Zhang, Qun Cui, Haiyan Wang

**Affiliations:** College of Chemical Engineering, Nanjing Tech University, Nanjing 210009, China; 201762100033@njtech.edu.cn (X.C.);

**Keywords:** SO_2_, H_2_S, adsorption breakthrough performance, competitive adsorption, Aspen adsorption simulation

## Abstract

The simultaneous adsorption and removal of low concentrations of SO_2_ and H_2_S using experimental and simulation methods were investigated in this paper. The adsorption breakthrough performance of the single-component SO_2_ or H_2_S was determined in the activated carbon fixed-bed test. Langmuir and extended Langmuir equations in the Aspen adsorption module were used to describe the adsorption equilibrium of the single and bi-component SO_2_ and H_2_S system, respectively. The effects of gas hourly space velocity (GHSV) and temperature on the dynamic adsorption process of the bi-component SO_2_/H_2_S system were investigated. The concentration distribution and adsorption capacity of SO_2_/H_2_S in the bed were simulated. The results showed that the simulation for the single-component breakthrough curves of SO_2_ or H_2_S agreed well with the experimental data. It indicated that the model and simulation yielded engineering acceptable accuracy. For the bi-component adsorption, the competitive adsorption effect was observed, with H_2_S as the weakly adsorbed component and SO_2_ as the strongly adsorbed component. The dynamic adsorption process showed the sequence of initial adsorption, breakthrough, replacement, and equilibrium. The breakthrough curves were characterized by the distinct hump (roll-up) for H_2_S, resulting from the replacement effect. The influence of GHSV and the temperature on the dynamic adsorption process were investigated, revealing that the lower velocity and temperature enhanced the adsorption. This work might be used for the design and optimization of adsorption bed for the simultaneous removal of SO_2_ and H_2_S in Claus tail gas.

## 1. Introduction

The Claus tail gas [1] typically contains low concentrations of SO_2_ (0.15–1.09%) and H_2_S (0.3–2.21%). It may exceed atmospheric emission standards and require additional removal treatment processes.

Currently, SCOT [2] and CANSOLV [3] processes are widely used for Claus tail gas treatment. However, these methods involve multiple complex steps, including reduction and oxidation reactions, rapid cooling, absorption, desorption, and incineration. It may increase the costs of energy consumption and operational costs. Schmidt [4] proposed an adsorption desulphurization process to replace the above processes of SCOT or CANSOLV, which effectively simplified the desulphurization process, improved the desulphurization accuracy, and reduced costs and energy consumption. The adsorption approach has drawn increasing attention regarding gas purification.

Previous research on the adsorption approach have focused on the removal of single-component SO_2_ [5,6,7,8,9,10,11,12,13] or H_2_S [14,15,16,17,18,19] individually, with limited studies addressing the simultaneous adsorption of bi-component gas [20,21]. The performance of activated carbon adsorbent ACS-1 for the Claus exhaust gas treatment was tested [22]. It demonstrated positive desulfurization efficiency for both H_2_S and SO_2_. The activated carbon adsorbent TL-1 [23] was tested to study the simultaneous adsorption of H_2_S and SO_2_ with the presence of CO_2_. The Cu–Ce–O modified activated carbon was tested to remove the 70 ppm H_2_S in blast furnace gas [24]. The adsorbed breakthrough time of H_2_S was extended if SO_2_ or O_2_ was included in the gas mixture and the introduction of SO_2_ could improve the adsorption of H_2_S on the modified activated carbon adsorbents. The simultaneous removal of H_2_S and SO_2_ not only enhanced the adsorbent utilization and the adsorption efficiency but also reduced the operational costs, highlighting its potential for industrial applications.

Analytical simulations were frequently used to evaluate the desulfurization capacity of various adsorbents. Mathematical models were used to predict breakthrough curves and kinetic models. For the 50 ppm SO_2_ adsorption over the palm kernel shell-activated carbon and xerogel adsorbent, the mathematical models, like Thomas, Yoon–Nelson, and Adam–Bohart models, were analyzed by curve fitting to determine the parameters. For the 70 ppm H_2_S adsorption over the modified activated carbon in the multicomponent blast furnace gas, five adsorption kinetics models were analyzed, while the Bangham model, including the pore diffusion effect, was selected for the best curve fitting to test data [24]. The over simplified mathematical models might result in several critical flaws, which were reported in the adsorption analysis of contaminated water [25]. Numerical methods, like the one-dimensional mass transfer balance determined by the partial differential equations, were frequently adopted with explicit input parameters. SO_2_ adsorption over the pistachio-nut-shell-activated carbon was simulated following the Langmuir isotherm model and the linear driving force kinetics model [11]. H_2_S physical adsorption in the fixed bed was simulated in COMSOL 5.4 software following the mass and energy balances [26]. For the multicomponent adsorption, several simulations reported the competitive adsorption phenomenon. The adsorption of SO_2_ and CO_2_ over the 41-S activated carbon adsorbent was simulated following the Langmuir and Ideal Adsorbed Solution Theory (IAST). The hump of the CO_2_ breakthrough curve, and the replacement of SO_2_ over CO_2_ predicted by the simulation were verified by test results [27]. The latest computational and experimental results showed the hump and selective adsorption in the CO_2_ and CH_4_ mixture [28]. Previous research provided fundamental knowledge and guided the adsorption simulation in this paper. However, little simulated research involving the bi-component simultaneous adsorption of SO_2_ and H_2_S were available, while further numerical studies were required.

In this study, the adsorption and breakthrough characteristics of SO_2_ and H_2_S over activated carbon fixed bed were investigated using a combination of experimental and simulated methods. The Langmuir and extended Langmuir equations in the Aspen adsorption module were applied to describe the adsorption equilibrium process of a single and bi-component SO_2_/H_2_S system, respectively, and compared with the experimental results. Furthermore, the effects of GHSV and temperature on dynamic adsorption characteristics of bi-component SO_2_ and H_2_S were investigated. The concentration distribution and adsorption capacity of SO_2_ and H_2_S within the activated carbon fixed bed were also examined. This work provides insights for the design and optimization of the adsorption bed for the simultaneous removal of SO_2_ and H_2_S in Claus tail gas, especially the increasing restrictions of the global air pollutant emission standards worldwide.

## 2. Experiments and Simulation

### 2.1. Experimental

**Figure 1:**
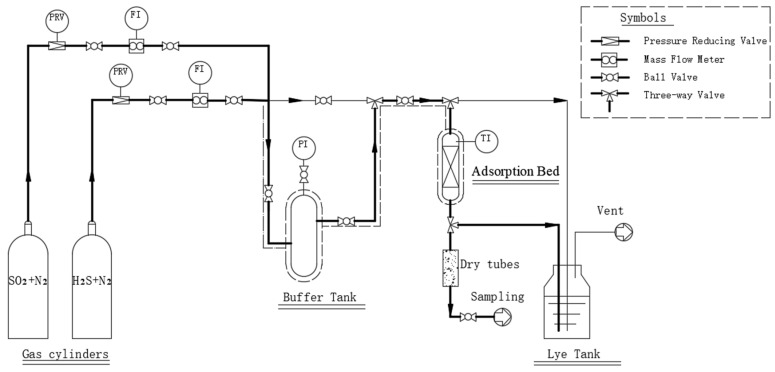
Schematic diagram of the simultaneous adsorption and removal of low concentrations of SO_2_ and H_2_S.

The adsorption bed (15 mm × 75 mm), packed with the activated carbon, was heated to the desired temperature using an electric heater (Shanghai Aore Electric Co., Ltd., Shanghai, China). N_2_ gas containing 1% SO_2_ or 2% H_2_S was introduced at controlled flow rates for the dynamic adsorption and desulfurization experiments. The sulfur content in the exhaust gas was continuously monitored at the bed outlet using the analytical instrument. When the sulfur concentration exceeded the breakthrough point, the experiment was terminated. The breakthrough point defined for SO_2_ was 100 ppm, and for H_2_S, it was 10 ppm in the experiment. It was close to the allowable emission levels which were 100 mg/m^3^ for SO_2_ and 10 mg/m^3^ for H_2_S, following the national air pollutant emission standards of China, like GB 31570-2015 [29] and GBZ 2.1-2019 [30]. The exhaust gas was then passed through the alkali tank for the final treatment. The schematic diagram of the adsorption desulfurization process is illustrated in Figure 1.

The bulk density of activated carbon was 450 kg/m^3^. The concentrations of H_2_S or SO_2_ in the desulfurized tail gas were measured by PGD3-IR gas analyzer from SSC (London, UK). The BET surface area of activated carbon was 1854 m^2^/g with the total pore volume of 0.97 cm^3^/g, including the micropore volume of 0.90 cm^3^/g, and the average pore diameter of 2.10 nm.

The dynamic adsorption experiments of SO_2_ or H_2_S were carried out under the adsorption conditions of 1% SO_2_ or 2% H_2_S, a GHSV of 173.32~866.58 1/h, and a temperature of 30 °C~155 °C.

### 2.2. Simulations

The single-component adsorption process of low-concentration SO_2_ or H_2_S over activated carbon was simulated by Aspen adsorption V11 [31]. The Langmuir adsorption isotherm equation was applied to model the adsorption bed, with the parameters of the adsorption bed (the diameter was 15 mm and the height was 75 mm) and the adsorbent being consistent with the experimental setup. The simulated breakthrough curves were compared with the experimental data to validate the isotherm model and its parameters.

For the bi-component adsorption, the simulation was conducted using the Extended-Langmuir isotherm model. The influences of GHSV and temperature on the breakthrough time of SO_2_ and H_2_S were analyzed to optimize the simultaneous and efficient removal ratio.

Aspen Adsorption simulation workflow was as follows. (1) Component Definition: specifying all the chemical components in Aspen Plus. (2) Model Transfer: importing component definitions to Aspen Adsorption environment. (3) Adsorption Bed Modeling: building adsorption bed modeling, the feed stream, and the product stream. (4) Parameters Input: inputting the parameters of the adsorption bed, the feed stream, and the product stream. (5) Solver Configuration, (6) Simulation Initialization, and (7) Execution and Analysis.

#### 2.2.1. Model Selection

The one-dimensional mass balance equation [9] for the fixed-bed adsorption is given in Equation (1).

(1) $$\frac{-D_{L}\partial^{2}C}{\partial z^{2}}+v\frac{\partial C}{\partial x}+\frac{\partial C}{\partial t}+\frac{1-\varepsilon_{b}}{\varepsilon_{b}}\rho_{p}\frac{\partial q}{\partial t}=0$$

The mass transfer due to the axial dispersion effect was usually ignored [10], as shown in Equation (2).

(2) $$\frac{{-D_{L}\partial }^{2}c}{\partial z^{2}}=0$$

The gas to solid surface mass transfer rate was determined by the Linear Driving Force (LDF) model, as shown in Equation (3).

(3) $$\frac{\partial q}{\partial t}=k_{f}\left(q^{*}-q\right)$$

Considering Equations (2) and (3), the mass balance Equation (1) could be expressed as Equation (4).

(4) $$\frac{\partial (\nu c)}{\partial x}+\frac{\partial c}{\partial t}+\frac{1-\varepsilon_{b}}{\varepsilon_{b}}\rho_{p}k_{f}\left(q*-q\right)=0$$

According to the Langmuir isotherm model in Aspen Adsorption, q* could be determined by Equation (5).

(5) $$q^{*}=\frac{{IP}_{1}*P_{i}}{1+{IP}_{2}*P_{i}}$$

For the extended Langmuir isotherm model used in the bi-component adsorption, the parameters were determined by Equations (6) and (7). Where, the subscripts *i* and *j* represented the two components, respectively.

(6) $$q_{i}^{*}=\frac{{IP}_{1i}*P_{i}}{1+({IP}_{2i}\cdot P_{i}+{IP}_{2j}\cdot P_{j})}$$

(7) $$q_{j}^{*}=\frac{{IP}_{1j}*P_{j}}{1+({IP}_{2i}\cdot P_{i}+{IP}_{2j}\cdot P_{j})}$$

In Aspen Adsorption, the partial differential Equation (4) was solved using the Upwind Differencing Scheme (UDS1), as shown in Equation (8).

(8) $$\frac{\partial q_{i}}{\partial t}=\frac{q_{i}-q_{i-1}}{△t}$$

The fluid velocity along the axial direction of the fixed bed was constant. The pressure drop along the axial direction of the adsorption bed was included using the Ergun equation to maintain the momentum balance. Since the diameter of the fixed bed was small and the adsorption was moderate, the temperature was assumed to be constant.

#### 2.2.2. Parameters for Simulation Calculations

The Langmuir isotherm parameters for SO_2_ and H_2_S adsorption on activated carbon at different temperatures were listed in Table 1. The adsorption process parameters were presented in Table 2.

## 3. Results and Discussion

### 3.1. Comparison of Single-Component Adsorption Experiments and Simulation

#### 3.1.1. SO_2_ Adsorption

**Figure 2:**
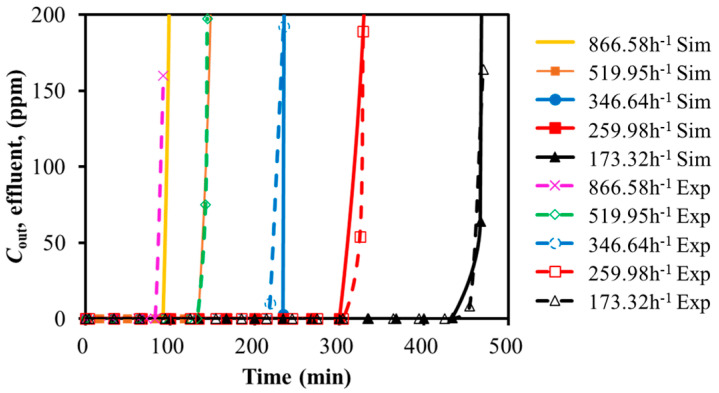
Calibration of breakthrough curves for SO_2_ with GHSV variation (30 °C).

**Figure 3:**
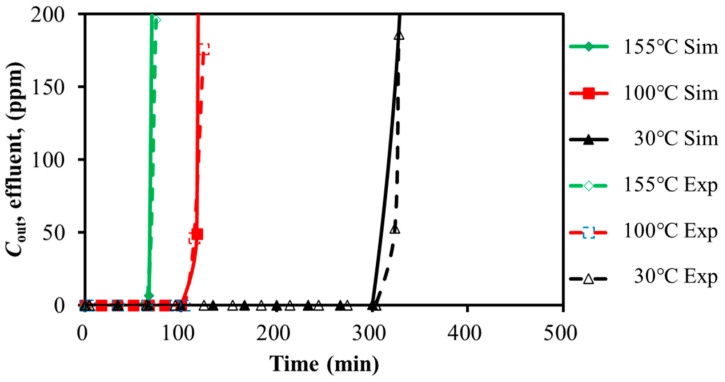
Calibration of breakthrough curves for SO_2_ with temperature variation (259.98 h^−1^).

The experimental and simulation results for SO_2_ adsorption on activated carbon at different GHSV and temperatures were compared in Figure 2 and Figure 3. The breakthrough time was summarized in Table 3 and Table 4. The horizontal axis represented the time. The vertical axis represented the gas volumetric concentration.

As shown in Figure 2, increasing the feed GHSV reduced the fluid residence time, leading to a shorter breakthrough time and lower adsorption efficiency. Similarly, Figure 3 demonstrated that the higher adsorption bed temperatures led to earlier breakthrough time. The experimental and simulated breakthrough time, as summarized in Table 3 and Table 4, showed acceptable approximation with the relative deviation of less than 6% and 3%. It might be acceptable within the scope of the engineering design. It seemed that the simulation was capable of representing the SO_2_ single-component adsorption system.

#### 3.1.2. H_2_S Adsorption

**Figure 4:**
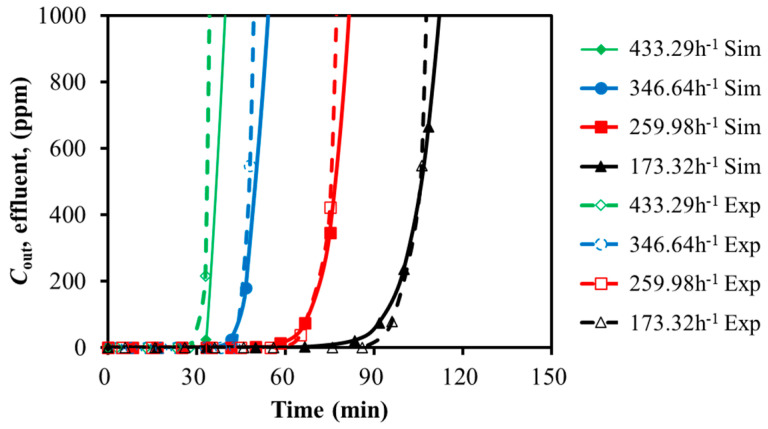
Calibration of breakthrough curves for H_2_S with GHSV variation (30 °C).

**Figure 5:**
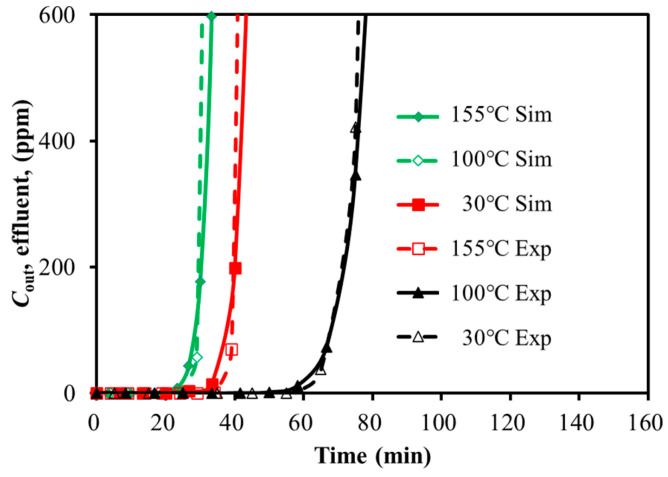
Calibration of breakthrough curves for H_2_S with temperature variation (259.98 h^−1^).

The experimental and simulation results for H_2_S adsorption on activated carbon by varying the feed GHSV and temperatures are presented in Figure 4 and Figure 5. The breakthrough times are compared in Table 5 and Table 6.

With the feed gas GHSV decreased by 50%, the breakthrough time was extended by 116.3% and 97.5% for H_2_S and SO_2_, respectively. With the temperature decreased by 80%, the breakthrough time was extended by 112.9% and 334.5% for H_2_S and SO_2_, respectively. It seemed that the low temperature and the slow GHSV were favorable for the adsorption. For the case of 30 °C, the breakthrough time of SO_2_ was five times of H_2_S, even though the feed concentration of H_2_S was twice that of SO_2_. SO_2_ showed stronger adsorption capacity over the activated carbon. It was efficient to adsorb SO_2_ with restricted temperature. However, the distinct adsorption capacity of H_2_S and SO_2_ over activated carbon might increase the difficulty of the simultaneous removal of the mixture. The adsorption performance of the mixture will be analyzed in the next section.

The predicted breakthrough times of H_2_S and SO_2_ were close to the test data with an acceptable error of less than 6.4%. It seemed that the simulation with the Langmuir isotherm model was able to approximate the adsorption of H_2_S and SO_2_ over activated carbon.

With the adsorption temperature increased from 30 °C to 155 °C, the error of simulation increased from 1.5% to 6.4% for H_2_S, and it increased from 0.9% to 5.4% for SO_2_. It seemed that the constant temperature assumption might result in larger prediction deviation. The same trend existed with the increased feed GHSV.

### 3.2. Simulation Calculation of Bi-Component Adsorption

#### 3.2.1. Effects of GHSV and Temperature on Dynamic Adsorption Characteristics

Using the parameters validated by the single-component adsorption simulation, the bi-component adsorption was modeled using the Extended-Langmuir isotherm model, as shown in Equations (6) and (7). The adsorption bed structure and adsorbent characteristics remained unchanged. The effects of GHSV and temperature on the bi-component adsorption breakthrough curves were also investigated. The breakthrough time for the bi-component adsorption was defined as the time when any component first reached its breakthrough concentration.

##### GHSV

**Figure 6:**
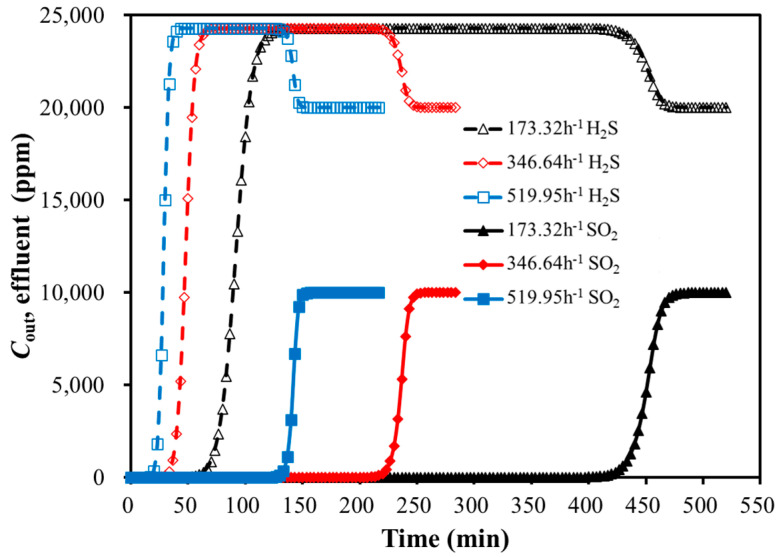
Breakthrough curves of bi-component adsorption with GHSV variation (30 °C).

Under the conditions of the adsorption temperature of 30 °C, a total pressure of 101 kPa, and the inlet gas concentrations of 2% H_2_S and 1% SO_2_, the breakthrough curves for the bi-component SO_2_ and H_2_S adsorption on the activated carbon were simulated with the feed GHSV variation. The results are presented in Figure 6 and Table 7.

As shown in Figure 6 and Table 7, the weakly adsorbed component H_2_S reached its adsorption saturation first during the bi-component adsorption of SO_2_ and H_2_S. It was followed by the characteristic hump in the breakthrough curve. This indicated that the competitive adsorption process might exist between SO_2_ and H_2_S. The strongly adsorbed component (SO_2_) was probably able to replace the weakly adsorbed component (H_2_S). With the development of the replacement, the two components reached the adsorption equilibrium. As a result, the concentrations at the bed outlet matched those at the inlet.

Aspen Adsorption simulated the replacement mechanism at the macro level, and the replacement mechanism was related to the characteristics of the adsorbent. SO_2_ on the adsorbent surface was adsorbed through van der Waals forces between its molecules and the π-electron layer of the adsorbent. The heat of the sublimation of SO_2_ (32.32 kJ/mol) was higher than that of H_2_S (23.01 kJ/ mol), meaning H_2_S had weaker adsorption on the adsorbent surface compared to SO_2_ [24]. Additionally, SO_2_ had greater polarity, leading to its preferential replacement of H_2_S.

For the bi-component SO_2_ and H_2_S adsorption, the higher GHSV resulted in a shorter breakthrough time. While the GHSV did not significantly affect the peak height of the H_2_S hump, it extended the duration of the hump. The impact of GHSV on the SO_2_ breakthrough curve was larger than H_2_S.

##### Temperature

**Figure 7:**
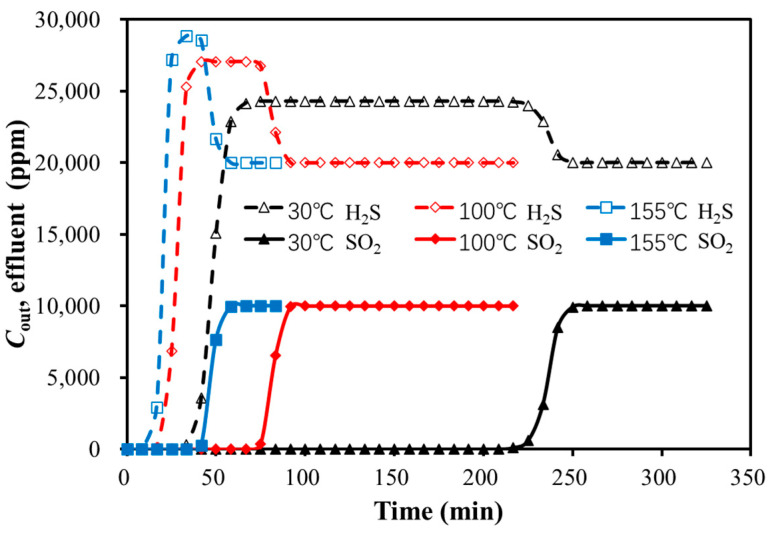
Breakthrough curves of bi-component adsorption with temperature variation.

The breakthrough curves for the bi-component SO_2_ and H_2_S adsorption on activated carbon were simulated at different temperatures, with a GHSV of 346.64 h^−1^, H_2_S concentration of 2%, SO_2_ concentration of 1%, and a total pressure of 101 kPa. The results are presented in Figure 7 and Table 8.

As shown in Figure 7 and Table 8, the peak height of the H_2_S hump increased with the rising temperature, while the duration of the hump decreased. The higher temperature led to a shorter breakthrough time for both SO_2_ and H_2_S. The effect of temperature was larger on the strongly adsorbed component (SO_2_) compared to the weakly adsorbed component (H_2_S).

#### 3.2.2. Comparison of Bi-Component and Single-Component Adsorption Breakthrough Time

The Extended Langmuir equation was the improvement upon the classical Langmuir model, used to describe complex systems involving the competitive multicomponent adsorption or adsorption on heterogeneous surfaces.

The breakthrough time for bi-component and single-component adsorption of H_2_S and SO_2_ were compared at the temperature of 30 °C and an GHSV of 346.64 h^−1^, as summarized in Table 9.

As shown in Table 9, the breakthrough time of the bi-component simulation was 10.5% and 18.2% earlier for SO_2_ and H_2_S, respectively. The bi-component adsorption led to an earlier breakthrough time for both SO_2_ and H_2_S compared to the single-component adsorption. This was attributed to the increase in feed species and the competitive interaction between the two gases, where the presence of one component reduced the available adsorption sites for the other.

#### 3.2.3. Concentration Distribution in the Adsorption Bed

**Figure 8:**
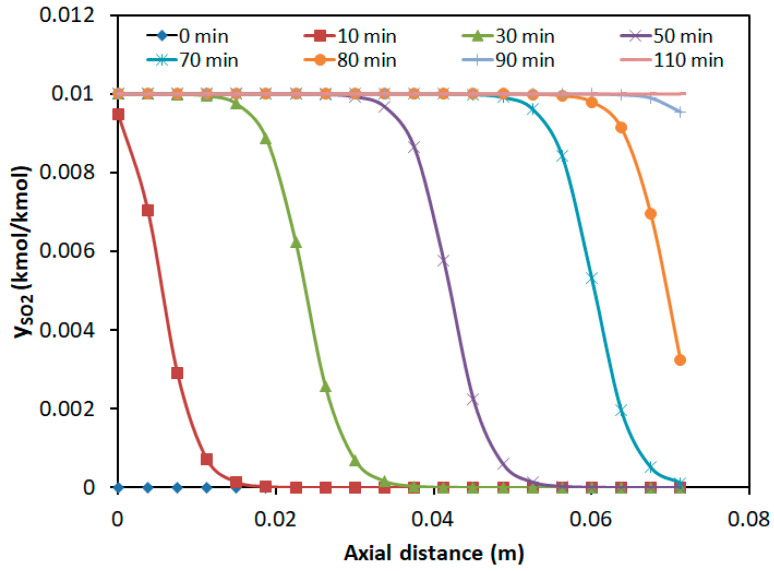
Axial concentration distribution of SO_2_ at different time intervals.

**Figure 9:**
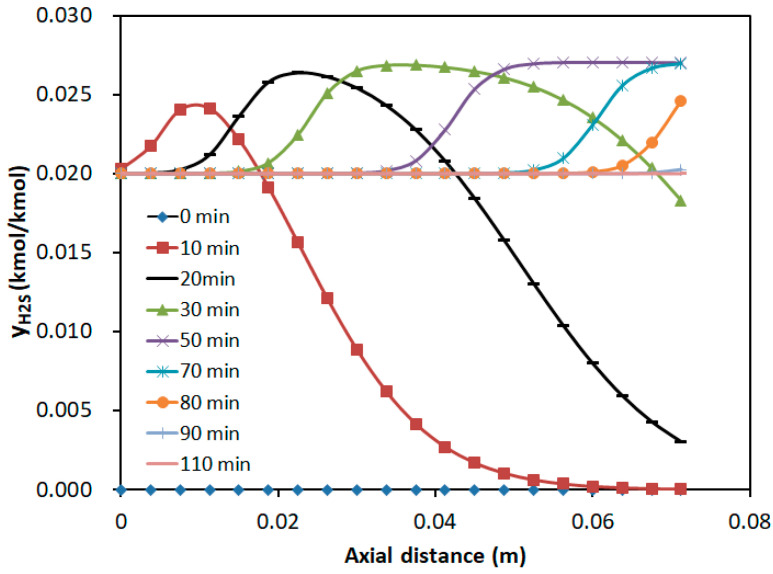
Axial concentration distribution of H_2_S at different time intervals.

Under the adsorption conditions of a H_2_S concentration of 2%, SO_2_ concentration of 1%, temperature of 30 °C, and a GHSV of 259.98 h^−1^, the axial concentration distributions of the two components in the adsorption bed at different time intervals are illustrated in Figure 8 and Figure 9. The horizontal axis represented the axial distance from the inlet of the adsorption bed, while the vertical axis indicates the concentration of SO_2_ and H_2_S in the gas mixture.

As shown in Figure 8, the SO_2_ mass transfer wave propagated forward and flattens over time. However, the breakthrough time was earlier, which increased adsorption resistance. At the bed outlet (*z* = 0.075 m), the SO_2_ concentration gradually increased from zero to the inlet concentration over time, with the breakthrough occurring at approximately 75 min and adsorption saturation reached at around 110 min. When H_2_S reached its breakthrough time at about 20 min, the bed above 0.03 m showed almost zero SO_2_ presence. The potential of over 60% of the fixed bed for the SO_2_ adsorption was not fully utilized.

Figure 9 demonstrated that during the bi-component adsorption, the hump appeared in the H_2_S concentration profile. This phenomenon arose from competitive adsorption, where H_2_S, as the weakly adsorbed component, was replaced by the strongly adsorbed SO_2_. As a result, the H_2_S concentration temporarily exceeded the inlet concentration, forming the hump. Over time, the hump propagated along the bed, and its height increased. At the bed exit (*z* = 0.075 m), the H_2_S concentration gradually rose from zero to the inlet concentration, with the breakthrough occurring at approximately 20 min and the adsorption saturation reached at around 110 min. It was consistent with the breakthrough curves.

#### 3.2.4. Distribution of Adsorption Amount in the Adsorption Bed

**Figure 10:**
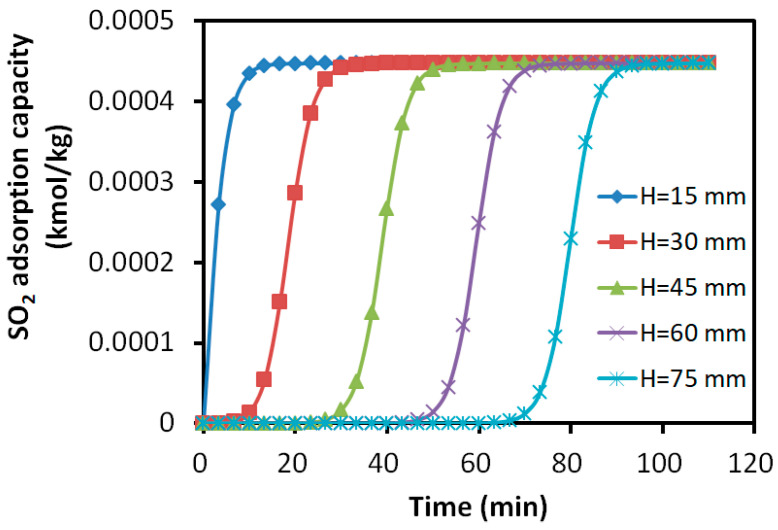
Variation in SO_2_ adsorption amount with time at different axial positions.

**Figure 11:**
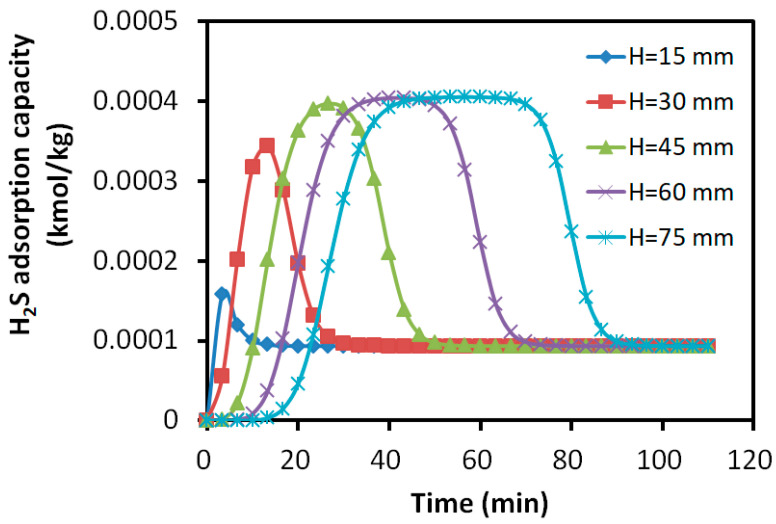
Variation in H_2_S adsorption amount with time at different axial positions.

The axial distribution of the adsorption amounts for the bi-component SO_2_ and H_2_S over the activated carbon are shown in Figure 10 and Figure 11. The horizontal axis represented the adsorption time, while the vertical axis indicated the adsorption amount of each component. The parameter H denotes the axial distance from the inlet of the adsorption bed.

The results revealed that the adsorption profiles of SO_2_ at different axial positions exhibited similar S-shapes, indicating the steady progression of the adsorption process along the bed. For the adsorption of H_2_S, the layers close to the inlet showed a slight increase in the adsorbed H_2_S. However, it was not able to maintain the S-shaped adsorption due to the replacement of SO_2_. The adsorbate rapidly decreased to the constant level of about 0.01% kmol/kg, which was about 25% of the peak value in the layers away from the inlet. For the layers close to the outlet, the peak adsorbed H_2_S was about 0.04% kmol/kg and it maintained a longer S-shape due to the later presence of SO_2_.

After the breakthrough of H_2_S at about 20 min, the adsorbed SO_2_ is mainly reserved within the bed layers of 0~30 mm, while H_2_S was mainly absorbed within the layers of 30~60 mm. The bed showed 40% of its desulfurization capability for SO_2_, while less than 40% for H_2_S due to the over 12.5% difference in the adsorption capacity peak. More than 20% of the bed was underutilized for the adsorption.

For the removal of the low-concentration SO_2_ and H_2_S, the simultaneous removal approach might be preferred to simplify the adsorption process and the equipment. However, it may require the extension of the bed due to the early breakthrough of H_2_S and the replacement effect. Water vapor and oxygen may be included to activate the reaction of H_2_S. As a result, the early breakthrough of H_2_S was delayed and the simultaneous removal performance might be improved.

The step-by-step approach might be optional since the early breakthrough and the replaced H_2_S could be adsorbed in the following unit without the influence of SO_2_. As a result, the potential adsorption capacity of SO_2_ might be maximized.

#### 3.2.5. Limitations of the Bi-Component Simulation

For the simulation of the bi-component adsorption of H_2_S and SO_2_ over the activated carbon, the extended Langmuir isotherm model, which was developed for the multicomponent adsorption, was adopted to predict the dynamic adsorption process of the trace fixed bed. The possible reaction of H_2_S and SO_2_ on the adsorbent surface were neglected since the low concentration, the low temperature, and the nitrogen mixture might suppress the reaction. However, the presence of water vapor and oxygen would activate the reaction, which was the case for the adsorbents with the atmosphere exposure experiences. The influences of chemical reactions require further experimental verification of the bi-component system. The distinct adsorption location of the bed indicating the variation of species concentrated on the adsorbents, and the breakthrough time gaps might be the evidence for the replacement effect in future experimental studies.

## 4. Conclusions

This paper investigated the simultaneous adsorption and removal of low concentrations of SO_2_ and H_2_S using experimental and simulation methods.

Test results showed the breakthrough time of SO_2_ was five times longer than H_2_S at 30 °C, and the activated carbon showed a better adsorption capacity over SO_2_. The decreased temperature and the low GHSV were favorable for the adsorption of both SO_2_ and H_2_S.

The simulation following the Langmuir isotherm model showed a less than 6.5% overestimation of the breakthrough time compared with the single-component test results. The simulation and the selected parameters were acceptable to reflect the adsorption of SO_2_ and H_2_S on the activated carbon.

The bi-component simulation following the extended Langmuir isotherm model predicted the hump breakthrough curves of H_2_S adsorption, which was probably due to the competitive adsorption of SO_2_ over H_2_S. It resulted in an 18% breakthrough time shortening of the system, which was controlled by the breakthrough of H_2_S. Additionally, the replacement of SO_2_ over H_2_S led to the distinct adsorption location over the fixed bed layers. The bed length should be extended to increase the simultaneous removal of the mixture.

Further research is required to investigate the chemical reaction effects on the bi-component adsorption, and to improve the simultaneous removal of SO_2_ and H_2_S by avoiding the unexpected early breakthrough of H_2_S.

## Figures and Tables

**Table 1:** Langmuir isothermal model parameters for SO_2_ [11] and H_2_S [32] at different temperatures.

Temperature (°C)	IP_1_ (kmol/kg/bar)		IP_2_ (bar^−1^)	
	SO_2_	H_2_S	SO_2_	H_2_S
30	1.410	0.056	890	21.1
100	0.300	0.031	474	17.2
155	0.134	0.023	319	15.5

**Table 2:** Adsorption process parameters of activated carbon fixed bed.

Adsorption Process Parameters	Units	Value
H_b_	m	0.075
D_b_	m	0.015
*ε*_*b*_	m^3^/m^3^	0.6774
*ρ*_b_	kg/m^3^	450
*R*_*P*_	(m)	0.125
S	-	0.91
*k_f_* of SO_2_ [31]	1/s	0.016
*k_f_* of H_2_S [31]		0.022

**Table 3:** Breakthrough time for SO_2_ with GHSV variation (30 °C).

Feed Gas GHSV (h^−1^)	Breakthrough Time (min)		Relative Deviation (%)
	Experimental	Simulation	
173.32	474	470	0.9
259.98	336	328	2.4
346.64	240	247	2.8
519.95	142	144	1.4
866.58	87	92	5.4

**Table 4:** Breakthrough time for SO_2_ with temperature variation (259.98 h^−1^).

Temperatures of the Adsorption Bed (°C)	Breakthrough Time (min)		Relative Deviation (%)
	Experimental	Simulation	
30	330	328	0.6
100	127	124	2.4
155	76	74	2.7

**Table 5:** Breakthrough time for H_2_S with GHSV variation (30 °C).

Feed Gas GHSV (1/h)	Breakthrough Time (min)		Relative Deviation (%)
	Experimental	Simulation	
173.32	93	89	4.5
259.98	65	64	1.6
346.64	43	44	2.3
433.29	31	33	6.1

**Table 6:** Breakthrough time for H_2_S with temperature variation (259.98 h^−1^).

Temperatures of the Adsorption Bed (°C)	Breakthrough Time (min)		Relative Deviation (%)
	Experimental	Simulation	
30	66	65	1.5
100	41	39	4.9
155	31	29	6.4

**Table 7:** Breakthrough time for bi-component SO_2_ and H_2_S adsorption with GHSV variation (30 °C).

Feed Gas GHSV (1/h)	Breakthrough Time (min)	
	H_2_S	SO_2_
173.32	59	431
346.64	36	221
519.95	10	134

**Table 8:** Breakthrough time for bi-component adsorption with temperature variation.

Temperatures of the Adsorption Bed (°C)	Breakthrough Time (min)	
	H_2_S	SO_2_
30	36	221
100	16	76
155	11	43

**Table 9:** Comparison of breakthrough time for bi-component and single-component adsorption.

Type	Breakthrough Time (min)	
	H_2_S	SO_2_
Bi-component	36	221
Single-component	44	247

## Data Availability

The original contributions presented in this study are included in the article. Further inquiries can be directed to the corresponding authors.

## Nomenclature

*C*	Concentration of adsorbate (kg/m^3^)
*C*_out, effluent_	Species concentration at the outlet (ppm)
y_SO2_	Dimensionless concentration of SO_2_ (kmol/kmol)
y_H2S_	Dimensionless concentration of H_2_S (kmol/kmol)
*v*	Flow rate of the fluid (m/s)
*z*	Axial direction of adsorption bed (m)
*ε_b_*	Bed porosity (-)
*ρ_p_*	Adsorbent particle density (kg/m^3^)
*ρ_b_*	Bed bulk density (kg/m^3^)
*ρ_s_*	Particle skeletal density (kg/m^3^)
*q*	Adsorption phase concentration (kg adsorbed/kg adsorbent)
*q**	Equilibrium adsorption capacity (kg adsorbed/kg adsorbent)
*k_f_*	Overall mass transfer coefficient (1/s)
*R_P_*	Particle radius of adsorbent (m)
*t*	Adsorption time (s)
H_b_	Height of adsorption bed (m)
D_b_	Adsorption bed inner diameter (m)
S	Spherical particle size of adsorbent (-)
*IP*_1_	Langmuir isothermal parameter (kmol/kg/bar)
*IP*_2_	Langmuir isothermal parameter (1/bar)
